# Possible Involvement of MyD88 in Regulating Stress Response in Mice

**DOI:** 10.3389/fnins.2021.621446

**Published:** 2021-03-12

**Authors:** Toru Hosoi, Yosuke Yamawaki, Hitomi Kimura, Shoko Honda, Koichiro Ozawa

**Affiliations:** ^1^Department of Clinical Pharmacology, Faculty of Pharmaceutical Sciences, Sanyo-Onoda City University, Yamaguchi, Japan; ^2^Department of Cellular and Molecular Pharmacology, Institute of Biomedical and Health Sciences, Hiroshima University, Hiroshima, Japan; ^3^Laboratory of Advanced Pharmacology, Daiichi University of Pharmacy, Fukuoka, Japan; ^4^Department of Pharmacotherapy, Graduate School of Biomedical and Health Sciences, Hiroshima University, Hiroshima, Japan

**Keywords:** myeloid differentiation primary response 88, brain-derived neurotrophic factor, corticosterone, immobilization stress, depression

## Abstract

Myeloid differentiation primary response 88 (MyD88) is an adapter protein of the toll-like receptor (TLR) family that regulates innate immune function. Here, we identified a novel role of MyD88 in regulating stress response. MyD88 deficiency decreased immobility time in the forced swim test without affecting locomotor activity in mice. Immobilization stress-induced production of serum corticosterone was also completely inhibited by MyD88 deficiency. Stress induced decrease in glucocorticoid receptor in the hippocampus. On the other hand, stress exposure in MyD88 deficient mice did not cause decrease in its level in the hippocampus. Furthermore, immobilization stress-induced reduction of brain-derived neurotrophic factor (BDNF) levels in the hippocampus was ameliorated by MyD88 deficiency. These results suggest that MyD88 deficiency attenuates depression-like behavior by regulating corticosterone and BDNF levels. Overall, these results indicate the key role of MyD88 in regulating stress response in mice.

## Introduction

Stress is one of key factor of depression and other mood disorders (Krishnan and Nestler, 2008). Genetic as well as environmental factors may contribute to the pathogenesis of such disorders (Nabeshima and Kim, 2013). However, the molecular mechanisms underlying this disorder are not yet completely understood. One of the pathophysiological responses to stress is the stimulation of the hypothalamic-pituitary-adrenal (HPA)-axis (Pariante, 2003). Stress exposure increases the production of corticotropin-releasing factor (CRF) in the rat hypothalamus (Imaki et al., 1992). CRF activates the HPA axis via the production of adrenocorticotropic hormone (ACTH) and corticosterone. Overproduction of corticosterone decreases the production of brain-derived neurotrophic factor (BDNF), a neurotropic factor involved in producing anti-depressant effect (Nestler et al., 2002). Indeed, it has been reported that immobilization stress decrease BDNF mRNA expression level through corticosterone in the rat hippocampus (Smith et al., 1995). Therefore, stress may stimulate the HPA axis, which may subsequently decrease BDNF expression level in the brain.

Increasing evidence suggests that inflammation is one of the mechanisms underlying these disorders (Raison et al., 2006; Dantzer et al., 2008). In one study, about 30% of human patients who underwent interferon therapy developed depression-like disorders (Loftis and Hauser, 2004). Furthermore, several cases of depression have been suggested to be accompanied by inflammation (Maes et al., 1995). In animal models, psychological stress caused by inescapable shock induced the production of proinflammatory cytokines in the rodent brain (Nguyen et al., 2000; O’Connor et al., 2003). Furthermore, interleukin (IL)-1β production in the brain in a chronic stress mouse model was found to play a key role in chronic stress-induced depression, and IL-1 receptor type 1 (IL-1R) knockout mice were not susceptible to these effects (Goshen et al., 2008; Koo and Duman, 2008). Serotonin is suggested to be involved in progression of depression and inhibitors of selective serotonin re-uptake inhibitors (SSRI) are widely used for the treatment of depression. Moreover, regulatory variation of serotonin transporters was suggested to affect depressive phenotype (Canli and Lesch, 2007). It has been reported that IL-1β increases activity of serotonin transporter in human JAR choriocarcinoma cells (Ramamoorthy et al., 1995) and increases serotonin neurotransmission (Shintani et al., 1993; Linthorst et al., 1994). Therefore, IL-1β may regulate serotonin metabolisms, which may be involved in progression of depression. Toll-like receptors (TLRs) are involved in activating innate immune function that can recognize components of microorganisms as well as endogenous ligands. Recently, repeated social defeat stress-induced social avoidance and anxiety were shown to be attenuated in TLR2- and 4-deficient mice (Nie et al., 2018). Additionally, lipopolysaccharide, one of the ligands of TLR4, has been reported to induce depression-like behavior in mice (Frenois et al., 2007). These results suggest that IL-1R- and TLR-mediated signaling play a key role in inducing psychological stress-related depressive symptoms. However, currently, the underlying molecular mechanisms by which inflammation-induced activation of IL-1R and TLR mediates the development of depressive symptoms are not well understood. Myeloid differentiation primary response 88 (MyD88) is an adapter protein commonly involved in regulating signal transduction under the action of TLR and IL-1 receptors (Muzio et al., 1997; Adachi et al., 1998; Kawai et al., 1999; Akira et al., 2001). Several pathophysiological functions of MyD88 in the CNS have been reported. It has been reported that brain development may be altered in MyD88-deficiency (Schroeder et al., 2021). Under the inflammatory condition, MyD88 signaling in the endothelial cells plays a role in neuronal activity (Gosselin and Rivest, 2008). MyD88 in the prefrontal cortex was increased in Alzheimer’s disease model mice, which may be involved in the disease progression (Rangasamy et al., 2018). Therefore, in the present study, we hypothesized that MyD88 plays a key role in the psychological stress response. We investigated the involvement of MyD88 in the stress-induced pathophysiological response in a model of mice deficient in MyD88.

## Materials and Methods

### Animals

Myeloid differentiation primary response 88-deficient mice were gift from Dr. Shizuo Akira (WPI Immunology Frontier Research Center, Osaka, Japan). The MyD88 deficient mice were generated at C57BL/6 mice. The mice were born healthy and developed normally until 20 weeks (Adachi et al., 1998). The information of the generation of the MyD88-deficient mice were indicated in previous report (Adachi et al., 1998). C57BL/6 control mice were purchased from Japan SLC, Inc. (Hamamatsu, Japan). Mice (3–5 mice/cage) were maintained in a pathogen-free facility at 22–24°C, with a 12-h light/dark cycle (lights on at 8:00 a.m., lights off at 8:00 p.m.). Food and water were given *ad libitum*. A hundred eight male mice (wild-type: 60, MyD88-deficient: 48, 8–12 week old) were used for the experiment. Different mice were used for any tests such as forced swim test, open-field test, measurement of corticosterone and quantitative PCR.

### Ethics Statement

Animal experiments were performed in accordance with the NIH Guide for the Care and Use of Laboratory Animals and approved by the Institutional Animal Care and Use Committee (IACUC: #A15-32) at Hiroshima University.

### Forced Swim Test

Control or MyD88-deficient mice were placed individually in a cylinder (diameter 11.5 cm, height 25 cm), which was filled with water (24 ± 1°C, height 17 cm). Then the movement of each mouse was recorded with digital video camera. It was monitored whether mice were drown or not in the place separated at least 3 m in same room. There were not drown, defined as sinking without moving, in this experiment. All the experiments were done during 23:30 to 24: 30. The periods of immobility time of each mouse were analyzed during 6 min. Immobility was defined as a lack of movement but included the presence of movements necessary to keep the head above water and it was manually measured.

### Measurement of Locomotor Activity

Open field tests were done for the measurement of locomotor activity. Control or MyD88-deficient mice were placed individually in a square box made up with clear acrylic board (48 cm × 48 cm × 48 cm) and measured movements of each mouse for 6 min using SCANET MV-10MT (MATYS TOYO SANGYO). All animals were habituated to the testing room for 20 min before the start of the session, and the testing sessions lasted for 6 min. The test room was dimly illuminated with indirect white lighting.

### Measurement of Corticosterone Levels

Mouse blood samples were collected immediately after stress exposure, and the samples were allowed to stand for 2 h at room temperature. Then, the samples were centrifuged at 3,000 rpm at 4°C for 15 min, and the supernatant serum was collected. The levels of corticosterone in the serum were measured at SRL, Inc.

### Immobilization Stress

Immobilization stress exposure had done based on previous report (Hosoi et al., 2019). The animals were laid on their back, and all four limbs of each mouse were corded and bonded to a board made of wood. Food and water were not available during stress exposure. For the control unstressed mice, four limbs were corded similar to the stressed mice, but not bonded to the board. Food and water were not provided to the control mice, similar to the stressed mice during the experiment. The samples were obtained 8 h after the immobilization stress.

### Tissue Isolation

The mice were killed by cervical spine fracture dislocation. Then, the brain was removed quickly. After removal, the tissue samples were washed with PBS. The hippocampus was dissected on an ice-cold plate. The samples were then snap-frozen in liquid nitrogen and stored at −80°C until use.

### Reverse Transcription

Tissue samples were homogenized in TriPure Isolation Reagent (Roche Diagnostics) using a polytron homogenizer. The total RNA was isolated according to the manufacturer’s instructions. Total RNA (2 μg) and the Oligo(dt)_12–18_ primers were incubated at 70°C for 10 min prior to reverse transcription, and cDNA was synthesized from the total RNA by reverse transcription using Superscript Reverse Transcriptase III (Invitrogen) and Oligo(dt)_12–18_ primer (Invitrogen) in 20 μL of reaction mixture containing First-Strand Buffer, 1 mM dNTP mix, 10 mM DTT, and RNase Inhibitor (20 U). After incubation for 1.5 h at 46°C, the reaction was terminated by incubating the cDNA at 70°C for 15 min.

### Quantitative PCR (qPCR) Analysis

Two-step quantitative PCR with THUNDERBIRD SYBR qPCR Mix (TOYOBO) was performed according to the manufacturer’s protocol using PikoReal 96 (Thermo Fisher Scientific). The cycling protocol was as follows: DNA polymerase activation at 95°C for 1 min, followed by denaturation at 95°C for 15 s, and annealing/extension at 60°C for 1 min, for 40 cycles. The relative value in each sample was calculated using the standard curve obtained from the non-treated hippocampus samples and normalized to GAPDH mRNA in the same samples. qPCR was performed using the following primers: GAPDH: upstream 5′ – AGG TCG GTG TGA ACG GAT TTG – 3′, downstream 5′ – GTA GAC CAT GTA GTT GAG GTC A – 3′; BDNF; upstream 5′ – GCC GCA AAC ATG TCT ATG AGG GTT – 3′, downstream 5′ – TTG GCC TTT GGA TAC CGG GAC TTT –3′; GR: upstream 5′ – CAA GGG TCT GGA GAG GAC AA – 3′, downstream 5′ – TAC AGC TTC CAC ACG TCA GC – 3′.

### Statistics

The results are expressed as mean ± SD. Statistical analyses were performed using Student’s *t*-test or two-way ANOVA followed by Tukey Kramer’s HSD test.

## Results

### MyD88 Deficiency Ameliorated Forced Swim-Induced Behavioral Despair

**FIGURE 1:**
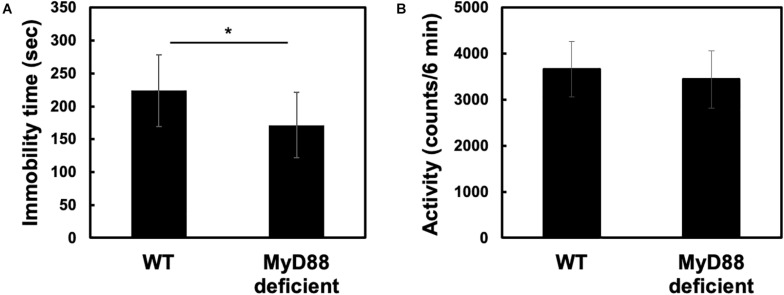
MyD88 deficiency decreased immobility time without affecting locomotor activity in the forced swim test. **(A)** Wild-type (WT) and MyD88-deficient (MyD88 deficient) mice were forced to swim for 6 min, and the immobility time was calculated. Values are presented as means ± SD. Student’s *t*-test: *p < 0.05; (WT: *n* = 12; MyD88 deficient: *n* = 12). **(B)** MyD88 deficiency did not affect locomotor activity. Locomotor activities (6 min) were measured for each genotype (WT and MyD88 deficient mice). Values are presented as means ± SD; Student’s *t*-test: not significance; (WT: *n* = 10; MyD88 deficient: *n* = 9).

The forced swim test (FST) is a widely used rodent model of depression-like behavior (Krishnan and Nestler, 2011). It is widely used to evaluate antidepressant action of drugs. FST causes immobility in mice and elicits a form of behavioral despair in them. To determine whether MyD88 is involved in promoting depression-like behavior, we subjected wild-type and MyD88-deficient mice to the FST. Interestingly, we found that the immobility time was significantly reduced in MyD88-deficient mice (WT vs. MyD88 deficient: 223.8 ± 54.71 vs. 171.4 ± 49.64) (Figure 1A). To determine whether the decrease in immobility time in MyD88-deficient mice was due to the increase in locomotor activity, we then performed an open-field test. We found that the locomotor activity of MyD88-deficient mice was not different from that of wild-type mice (WT vs. MyD88 deficient: 3660.8 ± 601.3 vs. 3443 ± 623.7) (Figure 1B). Therefore, these results suggest that the decrease in immobility time in MyD88-deficient mice was not due to an increase in locomotor activity. In addition, MyD88-deficiency did not affect body weight (Hosoi et al., 2010). Therefore, these results show that MyD88 may be involved in inducing depression-like behavior.

### MyD88 Deficiency Ameliorated Immobilization Stress-Induced Production of Corticosterone

**FIGURE 2:**
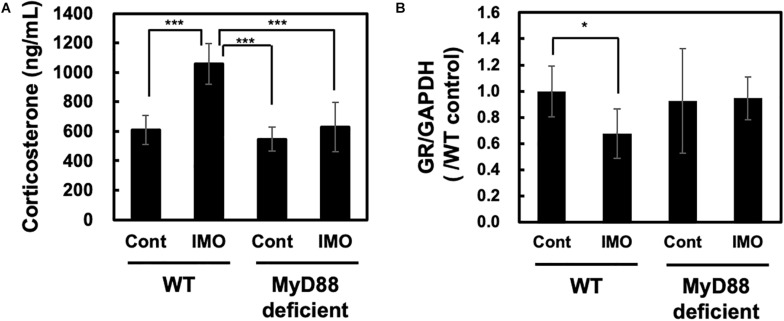
Corticosterone and glucocorticoid receptor levels in MyD88-deficient mice exposed to immobilization stress. **(A)** Immobilization stress-induced production of corticosterone was impaired in MyD88-deficient mice. Serum corticosterone levels in wild-type (WT) and MyD88-deficient (MyD88 deficient) mice were measured 8 h after the immobilization stress. Values are presented as means ± SD. Two-way ANOVA with Tukey’s honestly significant difference *post hoc* comparison was performed. The results of two-way ANOVA were as follows: immobilization: *F*(1) = 37.990 *p* < 0.0001; genotype: *F*(1) = 32.550, *p* < 0.0001; immobilization × genotype interaction: *F*(1) = 17.839, *p* = 0.0002. The result of *post hoc* test were as follows: ***p < 0.001, WT-cont vs. WT-IMO; WT-IMO vs. MyD88 deficient-cont; WT-IMO vs. MyD88 deficient-IMO; (WT-cont: *n* = 9; WT-IMO: *n* = 9; MyD88 deficient-cont: *n* = 8, MyD88 deficient-IMO: *n* = 9). **(B)** Expression levels of glucocorticoid receptor (GR) mRNA in MyD88-deficient mice did not change after exposure to immobilization stress. Hippocampus samples were obtained 8 h after exposure to immobilization stress (IMO) and subjected to real-time PCR analysis. Values are presented the ratio to WT control group as means ± SD. Two-way ANOVA with Tukey’s honestly significant difference *post hoc* comparison was performed. The results of two-way ANOVA were as follows: immobilization: *F*(1) = 2.801, *p* = 0.106; genotype: *F*(1) = 1.154, *p* = 0.293; immobilization × genotype interaction: *F*(1) = 3.595, *p* = 0.069. The result of *post hoc* test were as follows: *p = 0.023, WT-cont vs. WT-IMO; (WT-cont: *n* = 10; WT-IMO: *n* = 10; MyD88 deficient-cont: *n* = 5, MyD88 deficient-IMO: *n* = 5).

As exposure to stress increases CRF production in rat (Imaki et al., 1992) and activates the HPA axis in rat (Kant et al., 1987), we wondered whether stress-induced activation of the HPA axis was altered in MyD88-deficient mice. To check this hypothesis, we investigated whether circulating corticosterone levels showed changes in mice exposed to immobilization stress. Serum corticosterone levels were measured 8 h after exposure to immobilization stress. We found that immobilization stress led to the generation of circulating corticosterone in wild-type mice (WT-control vs. WT-IMO: 609.8 ± 99.0 vs. 1057.5 ± 139.6). This effect was completely absent in MyD88-deficient mice (WT-IMO vs. MyD88 deficient-IMO: 1057.5 ± 139.6 vs. 629.5 ± 166.8) (Figure 2A).

Production of corticosterone drives the activation of negative feedback mechanisms through the glucocorticoid receptor (GR). GR function has been reported to be decreased in depression (Holsboer, 2000; O’Connor et al., 2003). It has been reported that GR levels were decreased in immobilization stressed rat (Chen et al., 2008). Therefore, we investigated the expression level of GR in the hippocampus of wild-type and MyD88-deficient mice. As assessed by qPCR, we observed slight reduction of GR level by immobilization stress (WT-IMO vs. WT: 0.679 ± 0.189 vs. 1.000 ± 0.194). However, we did not observe difference in the expression levels of GR in control and immobilization stress at MyD88-deficient mice (MyD88 deficient-IMO vs. MyD88 deficient: 0.946 ± 0.164 vs. 0.926 ± 0.164) (Figure 2B).

### Stress-Induced Reduction in the Levels of Brain-Derived Neurotrophic Factor Was Ameliorated by MyD88 Deficiency

**FIGURE 3:**
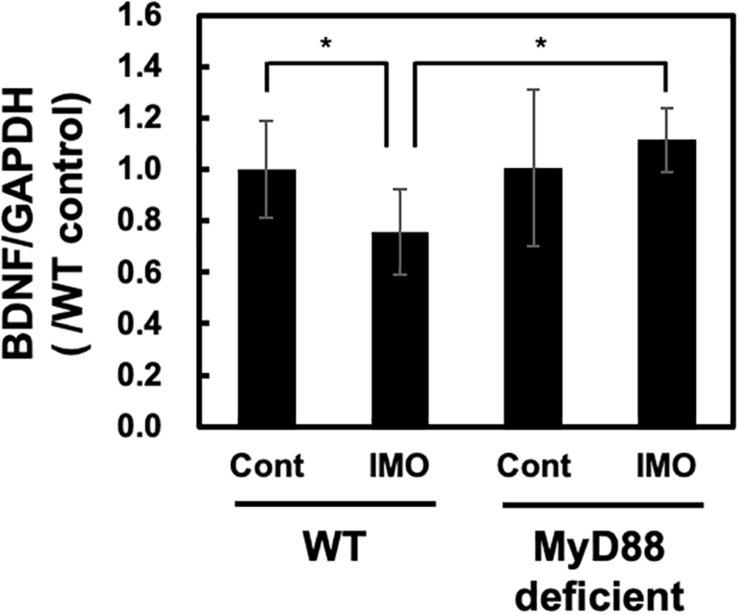
BDNF levels in MyD88-deficient mice exposed to immobilization stress. Immobilization stress-induced reduction in BDNF mRNA level was ameliorated in MyD88-deficient mice. Hippocampus samples were obtained 8 h after exposure to immobilization stress and subjected to real-time PCR analysis. Values are presented the ratio to WT control group as means ± SD. Two-way ANOVA with Tukey’s honestly significant difference *post hoc* comparison was performed. The results of two-way ANOVA were as follows: immobilization: *F*(1) = 0.766, *p* = 0.389; genotype: *F*(1) = 5.7515, *p* = 0.024; immobilization × genotype interaction: *F*(1) = 5.391, *p* = 0.028. The result of *post hoc* test were as follows: *p = 0.047, WT-cont vs. WT-IMO, *p = 0.128, WT-cont vs. deficient-IMO (WT-cont: *n* = 10; WT-IMO: *n* = 10; MyD88 deficient-cont: *n* = 5, MyD88 deficient-IMO: *n* = 5).

Hippocampal atrophy has been observed in patients with depression (Campbell et al., 2004). This hippocampal atrophy was reported to be accompanied by the inhibition of neurogenesis caused by decreased production of BDNF (Schmidt and Duman, 2007). On the other hand, increased production of glucocorticoids in response to psychological stress will also decrease BDNF production. Immobilization stress reduces BDNF mRNA levels in the rat hippocampus (Smith et al., 1995). Therefore, we examined the BDNF levels in the hippocampus after exposure to immobilization stress in wild-type and MyD88-deficient mice. As shown in Figure 3, we observed a fall in the BDNF mRNA levels after immobilization stress in the hippocampus of the wild-type mice, as assessed by qPCR analysis. This inhibitory effect on BDNF mRNA expression was absent in MyD88-deficient mice (WT-IMO vs. MyD88 deficient-IMO: 0.757 ± 0.168 vs. 1.116 ± 0.123) (Figure 3). Therefore, these results suggest that psychological stress-induced reduction in BDNF transcription is mediated by MyD88.

## Discussion

In the present study, we investigated the possible involvement of MyD88 in the development of depressive behavior and in regulating the psychological stress response in mice. We found that depressive behavior was significantly decreased in MyD88-deficient mice subjected to the FST. This result suggests that MyD88-deficient mice could be resistant to depression. Therefore, we further examined the possible role of MyD88 in stress-induced action. CRF activates the HPA axis through the production of ACTH and corticosterone. Moreover, stress exposure increases CRF production (Imaki et al., 1992) and stimulates the HPA axis in rats (Kant et al., 1987). Therefore, to analyze the possible role of MyD88 on stress-induced activation of the HPA axis, we measured the levels of corticosterone in mice exposed to immobilization stress. We found that immobilization stress-induced production of corticosterone was drastically attenuated in MyD88-deficient mice. Interestingly, we found that immobilization stress-induced reduction in BDNF levels in the hippocampus was ameliorated in MyD88-deficient mice. Corticosterone acts on GR expressed in the hippocampus and induces neuronal cell death (Behl et al., 1997). Furthermore, corticosterone has been reported to reduce BDNF production (Nestler et al., 2002). Therefore, decreased production of corticosterone in MyD88-deficient mice may contribute to the suppression of BDNF expression in the hippocampus. Overall, psychological stress in the brain may stimulate the HPA axis through the MyD88-dependent pathway. Production of corticosterone through activation of the HPA axis may reduce BDNF mRNA expression via the GR in the hippocampus. In consistent with previous report (Chen et al., 2008), brain GR level was decreased in immobilization stress (Figure 2B). On the other hand, GR level was not affected in MyD88-deficient mice (Figure 2B). Early life stress was suggested to be involved in decreased GR expression through epigenetic changes of GR promoter (Seo et al., 2020). Therefore, possible involvement of MyD88 on epigenetic mechanisms and GR expression may be required to be analyzed in the future experiments. The reduction in BDNF production may be associated with vulnerability to psychological stress, as BDNF is an important neurotropic factor involved in neuronal survival (Hofer and Barde, 1988). Therefore, MyD88 deficient mice may resist against stress through regulating HPA axis and BDNF level in the hippocampus.

We performed FST analysis for the measurement of depression. However, the results of FST analysis would be affected by the secondary effect of gene-deficiency of MyD88. For instance, it would be possible that lack of hand-eye coordination in MyD88 deficient mice may reduce swimming, rather than attenuation of depression. However, as far as we know, there is no literature indicating that gene-deficiency of MyD88 would affect hand-eye coordination. Furthermore, MyD88 deficiency do not affect body weight (Hosoi et al., 2010), suggesting that the effect of FST movement may not be affected by the secondary effect of body weight. On the other hand, it has been reported that MyD88-deficiency affected food intake and body temperature under condition of lipopolysaccharide (LPS)-induced inflammation (Ogimoto et al., 2006). Therefore, MyD88 may be involved in food intake and body temperature under the LPS-induced inflammatory condition. Also, it has been reported that neuropathic pain was reduced at MyD88 deficiency in sensory neurons (Liu et al., 2016). Therefore, MyD88 may affect some physiological outcome in the specific situations, which we think that these physiological outcomes may not affect current behavioral changes of FST.

Proinflammatory cytokines such as IL-1 and TLRs have been suggested to be involved in the pathogenesis of depression (Raison et al., 2006; Nie et al., 2018). However, the underlying molecular mechanisms of inflammation-induced depression are not well understood. We found that MyD88 may play a role in mediating psychological stress in the brain and may be a key molecule in the linkage between inflammation and depression. In addition to the TLRs roles for immune function, they are also expressed in non-immune cells and involved in development (Anthoney et al., 2018). In CNS, several reports suggested that TLRs are involved in axonal growth, neurogenesis and structural plasticity (Okun et al., 2011). For instance, TLR2 and TLR4 are expressed in neural progenitor cells (NPCs) and suggested to affect its proliferation through MyD88 (Rolls et al., 2007). It has been suggested that MyD88-deficiency will enhance NPC proliferation (Rolls et al., 2007). Decreased neurogenesis has been suggested to be involved in depression (Eisch and Petrik, 2012). It has been reported that stress exposure to primates will decrease neurogenesis, which was reversed by antidepressants (Czéh et al., 2001). Therefore, it may also be possible that defective MyD88 signaling would increase neurogenesis, which may affect depression. Further analysis may require to assess these possibilities.

The current results highlight MyD88 as a novel pharmacological target for treating depression. Therefore, our results provide novel insights into the mechanisms of and treatment strategies for psychological stress-related disorders.

## Data Availability Statement

The original contributions presented in the study are included in the article, further inquiries can be directed to the corresponding authors.

## Author Contributions

TH designed the study. HK, YY, and SH performed the experiments. TH and YY wrote the manuscript. TH, YY, and KO interpreted the results and edited the manuscript. All authors contributed to the article and approved the submitted version.

## Conflict of Interest

The authors declare that the research was conducted in the absence of any commercial or financial relationships that could be construed as a potential conflict of interest.
